# Linear models of activation cascades: analytical solutions and coarse-graining of delayed signal transduction

**DOI:** 10.1098/rsif.2016.0409

**Published:** 2016-08

**Authors:** Mariano Beguerisse-Díaz, Radhika Desikan, Mauricio Barahona

**Affiliations:** 1 Department of Mathematics, Imperial College London, London SW7 2AZ, UK; 2 Department of Life Sciences, Imperial College London, London SW7 2AZ, UK

**Keywords:** activation cascades, model reduction, ordinary differential equation models, signal transduction

## Abstract

Cellular signal transduction usually involves activation cascades, the sequential activation of a series of proteins following the reception of an input signal. Here, we study the classic model of weakly activated cascades and obtain analytical solutions for a variety of inputs. We show that in the special but important case of optimal gain cascades (i.e. when the deactivation rates are identical) the downstream output of the cascade can be represented exactly as a lumped nonlinear module containing an incomplete gamma function with real parameters that depend on the rates and length of the cascade, as well as parameters of the input signal. The expressions obtained can be applied to the non-identical case when the deactivation rates are random to capture the variability in the cascade outputs. We also show that cascades can be rearranged so that blocks with similar rates can be lumped and represented through our nonlinear modules. Our results can be used both to represent cascades in computational models of differential equations and to fit data efficiently, by reducing the number of equations and parameters involved. In particular, the length of the cascade appears as a real-valued parameter and can thus be fitted in the same manner as Hill coefficients. Finally, we show how the obtained nonlinear modules can be used instead of delay differential equations to model delays in signal transduction.

## 1. Introduction

**Figure 1.**
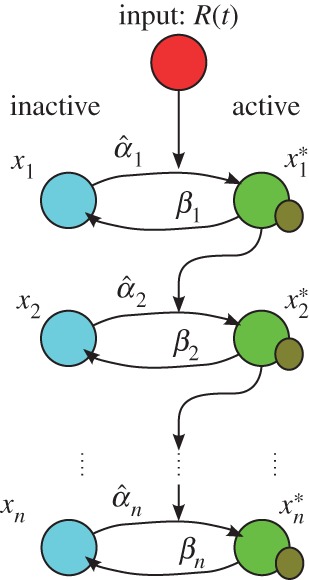
A typical protein activation cascade of length *n*. The proteins (nodes) in the cascade can either be in an inactive (*x_i_*) or active (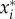) state. An external signal *R*(*t*) activates the first node. Once a node is active, it activates the next component in the cascade until the end. The activation rate of each *x_i_* is *α_i_*, and the deactivation rate of each 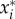 is *β_i_*. Adapted from [1]. (Online version in colour.)

Activation cascades are pervasive in cellular signal transduction systems [1,2]. In its simplest form, an activation cascade comprises a set of components (typically proteins) that become sequentially activated in response to an external stimulus (figure 1). These systems have been the subject of numerous studies, experimental and theoretical [1,3–9]. The role of activation cascades in cellular signal transduction is manifold. Cascades can relay, amplify, dampen or modulate signals in order to achieve a variety of cellular responses. One of the best-studied examples of such a system is the *mitogen-activated protein kinase* (MAPK) cascade, which plays a central role in key cellular functions, such as regulation of the cell cycle, stress responses and apoptosis [2].

Models of activation cascades are known to exhibit a range of nonlinear behaviours, including ultrasensitivity [6,10] and multistability [5,11]. Linearized models of cascades [1] (the so-called ‘weakly activated’ regime studied here) are also of theoretical interest, and have been studied to evaluate signalling times [12], signal specificity [13] and optimal gain [4]. Such linearized descriptions of cascades often appear as part of larger and more complicated models, and have been shown to be useful in model reduction techniques [14]. Hence obtaining coarse-grained representations of such cascades would be useful not only to simplify their mathematical analysis but also computationally, to allow for compact implementations in models for systems biology. Furthermore, weakly activated cascades are of importance in quantitative biology as they have been observed experimentally [15]. In this context, it would be desirable to estimate the length of an unobserved cascade from data without having to create and fit several models, each with a different number of equations to represent the varying length of the cascade.

Here, we present a study of analytical solutions of ordinary differential equation (ODE) models of linear activation cascades. First, we obtain general solutions for weakly activated cascades. We then focus on the case when the gain of the cascade is optimal (i.e. when all deactivation rates are identical), and find that a lower incomplete gamma function with only three real-valued parameters represents the output of the entire cascade. We exemplify the use of this coarse-grained solution to describe the downstream output induced by several time-dependent inputs of interest, including step functions, exponentially decaying signals, Gaussian inputs and periodic stimuli. We also show that the obtained solution has real-valued parameters directly linked to the length and filtering properties of the cascade, and can thus be used to fit data capturing efficiently the delay and distortion introduced by the cascade. We also explore the application of our results to non-optimal cascades, i.e. when the requirement of identical deactivation rates is relaxed. When only one deactivation rate is different, the equations can be reordered, so that a lumped gamma function representation can be used for the block of identical proteins without altering the final output of the cascade. We also show that when the deactivation rates are randomly distributed, the gamma function can still be used to represent the distribution of the outputs of the cascade. Finally, we show how the gamma function representation of a cascade can be used as a computationally efficient replacement of delay differential equations (DDEs).

## 2. Weakly activated cascades and their gamma function solution

Consider a cascade involving *n* components that are activated in succession. Upon perception of the input signal 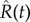, the first inactive component (*x*_1_) is transformed into its activated form (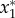), which then activates the next component (*x*_2_). Sequential activation of *x_i_* by 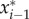 continues until the end of the cascade. The output of the cascade of length *n* is the activated form of the last component, 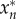. In the case of the MAPK cascade, the components are proteins, and the activation corresponds to a post-translational modification, i.e. phosphorylation. However, the formalism can also describe other sequential biochemical processes with similar functional relationships, e.g. *n*-step deoxyribonucleic acid (DNA) unwinding [16].

If we use mass-action kinetics without an intermediate complex to describe protein activation, the reaction describing the activation of *x*_1_ is
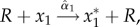
and for the rest of the proteins *x_i_* (*i* = 2, … ,*n*) we have
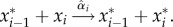
We also assume that all proteins deactivate spontaneously with constant rate
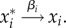
The system of nonlinear ODEs describing the time evolution of the full activation cascade is [1]2.1
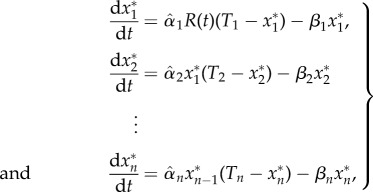
where we have defined the total amount of each protein 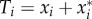, so that the inactive form is 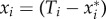. We also assume that the model operates over time scales where there is no significant protein production, so that the amount of each protein *T_i_* can be considered constant. If the time scales are such that the total amount of protein varies significantly, then each *T_i_* would have to be described by its own ODE according to additional biological knowledge.

### 2.1. The general solution for weakly activated cascades

As shown in [1], in the *weakly activated* regime 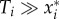, one takes the approximation 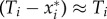, and the original system (2.1) can be rewritten as a driven linear system:2.2
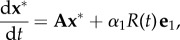
where 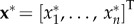, 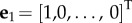 is the first *n*×1 vector of the canonical basis, and the *n* × *n* rate matrix **A** is2.3
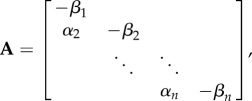
where 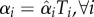.

This system can be solved using the Laplace transform with auxiliary variable *s*. If the cascade receives an integrable input *R*(*t*), it is easy to show that the Laplace transform of the *k*th protein is2.4
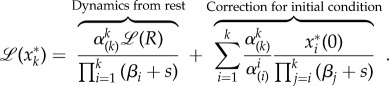
The first term on the right-hand side corresponds to the Laplace transform of 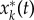 for initial conditions 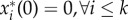 (i.e. the cascade starts from rest), and the second term contains the correction for non-zero initial conditions. The term *α*_(*k*)_ is the geometric mean of the activation rates up to *k*:2.5
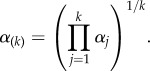
Note that if 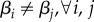, then
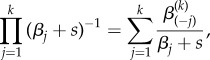
where
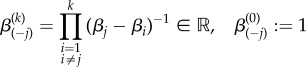
is a constant that depends only on the deactivation rates. Now we can express equation (2.4) as2.6
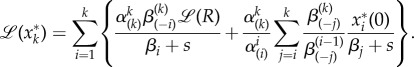
Using linearity and the convolution properties of the Laplace transform, the output of the cascade is finally obtained as2.7
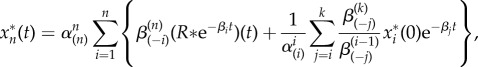
where
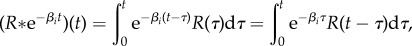
and the pre-factor incorporates the product of all the activation rates,
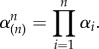
Although equation (2.7) describes the evolution of a general initial condition, in this study we will assume henceforth that the cascade is initially fully inactive (i.e. 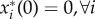). In the cases when 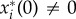, then the exponential correction introduced by the initial conditions can be incorporated to the calculations.

**Example 2.1.**

If a linear cascade is subject to a constant stimulus given by the step function 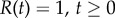, and 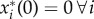, equation (2.7) shows that the output of the last protein in the cascade is given by2.8
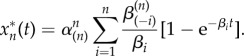

### 2.2. Optimal linear cascades

Activation cascades are substantial modules of the cell-signalling machinery and, as such, they should be efficient in minimizing the use of energetic resources, such as adenosine triphosphate, or of cellular building blocks, such as amino acids. In the study of Chaves *et al*. [4], it was shown that when a weakly activated cascade (2.2) is required to provide a given gain, the amplification is achieved optimally when the number of steps in the cascade (e.g. the number of proteins) is finite and all deactivation rates are equal, i.e. 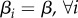. This result means that arbitrarily long cascades are not useful for cells when a particular amplification gain from external signals is required. For an optimal cascade, the rate matrix in equation (2.2) becomes2.9
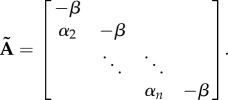


## 3. Linear cascades under different input functions

We now consider the time-dependent output of a cascade under four different inputs of biological interest.

### 3.1. Step-function stimulus

In an experimental setting, one often studies the response of a biological system to a step-function stimulus such as constant temperature, light or treatment started at time *t* = 0. In this case, the stimulus is
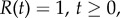
and the solution to (2.2) with initial condition 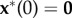 is3.1
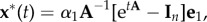
where **I***_n_* is the *n* × *n* identity matrix, and 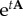 is the matrix exponential.

If the cascade is optimal (i.e. 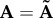), the Laplace transform of the last protein given by (2.4) becomes
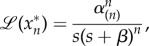
and taking the inverse transform we obtain3.2
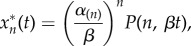
where3.3
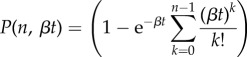
is the normalized lower incomplete gamma function whose general form is [17]3.4
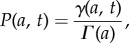
where *Γ*(*a*) is the gamma function and
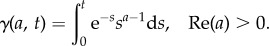


### 3.2. Exponentially decreasing stimulus

When the first protein in the cascade is subject to an exponentially decaying stimulus (e.g. when the input is a reactive molecule or a molecule that becomes metabolized, or if the receptors become desensitized)
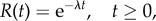
then the solution to (2.2) with initial condition 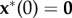 is3.5
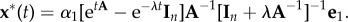
If we assume that the cascade is optimal (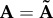), then
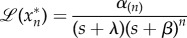
and the output of the cascade is given by3.6
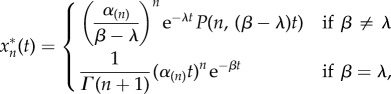
where *α*_(*n*)_ is defined in (2.5). As in the case of constant stimulus, the solution is also given in terms of the lower incomplete gamma function.

### 3.3. Periodic stimulus

In certain experimental settings, we are interested in the response of a system to a periodic stimulus, e.g. circadian rhythms or day/night cycles [18]. Let us consider a linear cascade of length *n* with periodic input
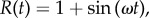
which oscillates between 0 and 2 with mean 1 and frequency *ω* > 0. From a resting initial condition, the solution to equation (2.2) is3.7
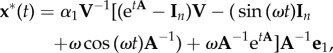
where 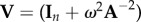.

When the cascade is optimal (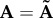), the explicit solution for the *n*th protein in the cascade is3.8
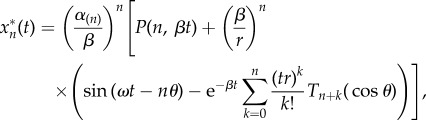
where 
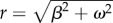
, 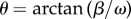 and the 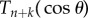 are the Chebyshev polynomials evaluated at cos*θ*.

Asymptotic limits provide useful insights. When the frequency is large compared with the deactivation rate, i.e. 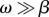, then 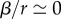, 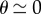 and we obtain
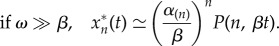


Hence for large frequencies the oscillations in equation (3.8) are filtered out, and the solution approaches the response to the step function given by equation (3.2). Conversely, when the deactivation of the proteins dominates the frequency (i.e. 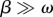) the behaviour of 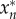 will be dominated by the sinusoidal input.

In general, asymptotically as *t* → ∞, the cascade acts broadly as a filter with an overall amplification 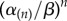, and an oscillatory term attenuated by a factor 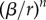 with a delay phase *nθ*:3.9
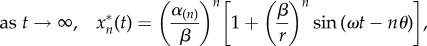
where we have used the fact that 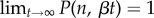. Note that 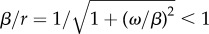, which implies that 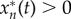 for all *t*. Cascades with more complicated temporal stimuli can be analysed similarly using the Fourier series expansion of *R*(*t*).

### 3.4. Gaussian stimulus

Gaussian input functions are employed to represent drug intake and other such signals. Consider a cascade of length *n* with input3.10
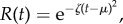
which describes a bell-curve centred at *t* = *μ*, with height 1 and amplitude *ζ*. The solution of equation (2.2) from inactive initial conditions under this input is then given by3.11
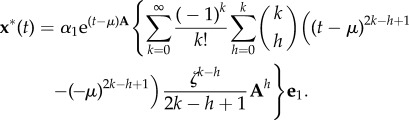


When a Gaussian input 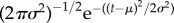 becomes increasingly narrow (i.e. *σ* → 0), it approaches in the limit a Dirac delta function: 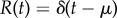. In that case, from equation (2.7) the solution for the *n*th protein is3.12
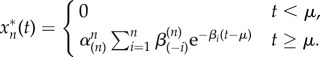


## 4. Applications of the analytical solutions to the coarse-grained modelling of cascades

### 4.1. Model simplification and parameter fitting

**Figure 2.**
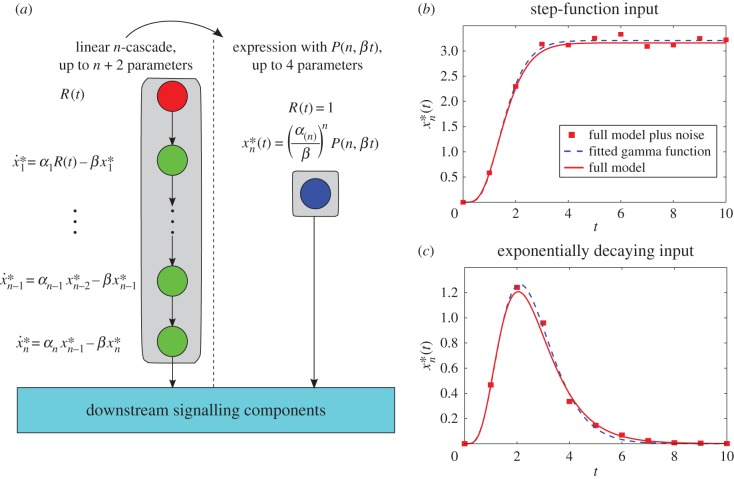
Simplification of a linear activation cascade and fitting with incomplete gamma functions. (*a*) Schematic of an optimal linear cascade (2.2) and its corresponding equivalent output function (3.2) under a step-function input. The output of the cascade, *x_n_*, relays the signal to downstream components of the pathway. Whereas the full *n*-dimensional model of the cascade has up to *n* + 2 parameters (*α_i_*, *β*, *n*), the condensed expression for the output has three parameters *α*_(*n*)_, *β*, *n*. Fitting time-courses of a cascade with two different inputs: (*b*) a step function and (*c*) an exponentially decaying stimulus. In both cases, we considered an optimal cascade with *n* = 5 components and parameters *α*_1_ = 3, *α_i_* = 4 for *i* = 2, … ,5, and *β* = 3. The step-function input was *R*(*t*) = 1, *t* ≥ 0 and the exponentially decaying input was 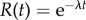 with *λ* = 1. The solid lines indicate the solutions to the full system of *n* ODEs. The squares are ‘noisy data’ generated from the full model: *x*_5_(*t*) sampled at *t* = {0, 1, … , 10} with additive Gaussian noise with standard deviation *σ* = 0.05. The dashed lines are fits of the noisy data using the corresponding incomplete gamma function expressions, equations (3.2) and (3.6). The fits were carried out using the squeeze-and-breathe algorithm [20]. (Online version in colour.)

The expressions of the cascade output, 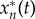, obtained in the previous sections can be used to fit activation data to a small number of parameters. Rather than fitting observations to an entire module of ODEs with 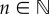 components, the expressions with the gamma function contain three parameters (*α*_(*n*)_, *β*, *n*) to describe an optimal cascade, and possibly other real parameters associated with the input (e.g. *λ* for the exponentially decaying input, or *ω* for the periodic stimulus). In particular, note that the first argument of the incomplete gamma function (3.4), which is linked to the cascade length, is a positive *real number* [19]. Hence when fitting data (figure 2), the estimated length of the cascade is turned into a real-valued parameter 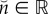, similar to what is done with Hill coefficients to represent multiple mechanistic steps [21].

In figure 2, we present the application of this approach to the fitting of the output of an optimal cascade with two different inputs. We start by generating simulated data from a cascade of length *n* = 5 with parameters *α*_1_ = 3, *α_i_* = 4, for *i* = 2, … ,5 (so that *α*_(*n*)_ = 3.776), and *β_i_* = *β* = 3, for *i* = 1, … ,5. One cascade is subject to a constant stimulus *R*(*t*) = 1 and the other to an exponentially decaying input 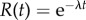 with *λ* = 1. We solve numerically the *n*-dimensional system of equation (2.2) for both inputs (solid lines in figure 2*b,c*), and then we generate ‘observations' by sampling the output *x*_5_(*t*) at times *t* = {0, 1, … , 10} with additive Gaussian noise drawn from 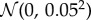. We consider these samples as our ‘noisy data’ (squares in figure 2*b,c*) and we fit the gamma function expressions (3.2)^1^ and (3.6), respectively, using a Matlab implementation of the squeeze-and-breathe evolutionary Monte Carlo method which is especially appropriate for time course series [20].^2^ The dashed lines in figure 2*b* show the fits to both cascade outputs, and the estimated values are close to the ‘true’ ones: for the constant stimulus cascade, the fitted values are 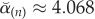, 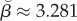 and 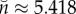; for the exponentially decaying stimulus, the estimated values are 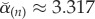, 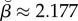, 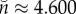 and 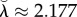.

### 4.2. Application to near-optimal cascades with random deactivation rates

Strict optimality of cascades [4] requires that all deactivation rates of the proteins be identical (i.e. *β_i_* = *β* for all *i*). Likewise, our expression for the cascade output in terms of the incomplete gamma function is only strictly valid under the same assumption. Naturally, it is unreasonable to expect identical rates in a biological system. Therefore, we ask the question: if we relax this condition and allow each *β_i_* to be an independent and identically distributed (iid) random variable with mean 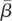, can we still approximate the output of the module with a gamma function?

**Figure 3.**
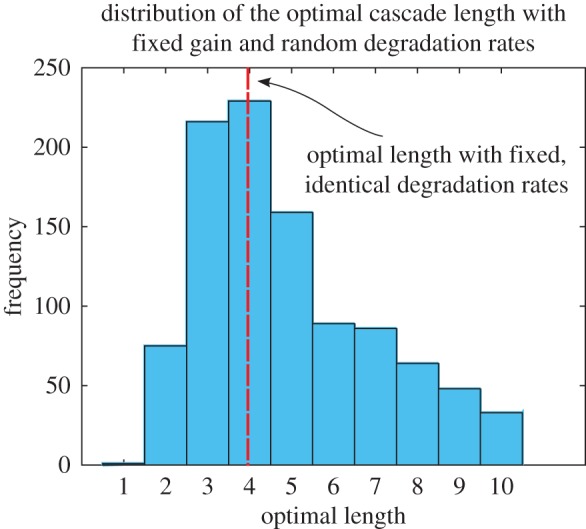
Distribution of optimal cascade lengths for non-identical (random) deactivation rates. Simulation of 1000 random sets of cascades with fixed expected gain *G* = 8 under a step-function of intensity *α*_1_ = 1.2 and *α_i_* = 1. The length of each cascade is grown from *n* = 1, … ,10 with random deactivation rates 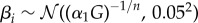, and the length of the cascade that achieves maximum amplification is recorded. The figure presents the histogram of the observed optimal lengths. (Online version in colour.)

**Figure 4.**
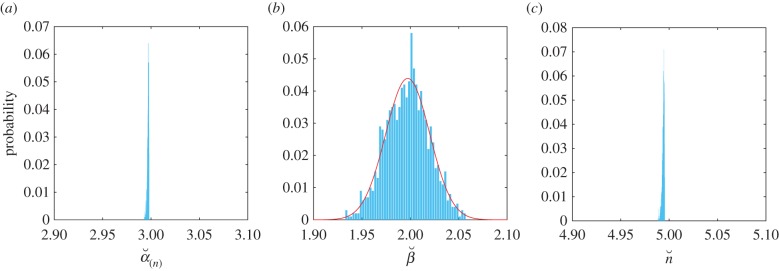
Estimation of parameters in random, near-optimal cascades. Distribution of estimated parameters 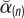, 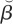 and 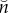 obtained when fitting equation (3.2) to 1000 cascades in which *α*_(*n*)_ = 3, *n* = 5 and 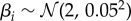 for *i* = 1, … ,5. The mean of the estimated parameters are: 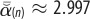, 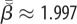 and 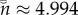. The curve shows a fitted normal distribution with mean 1.997 and variance 0.022^2^. (Online version in colour.)

**Figure 5.**
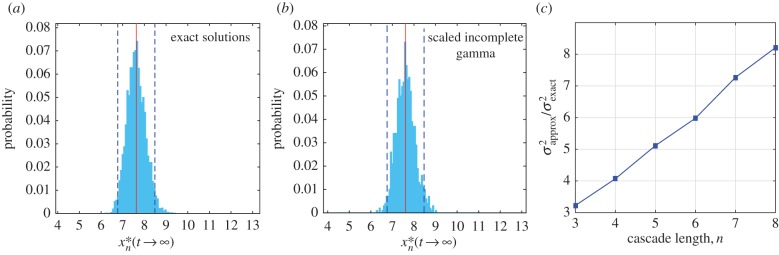
Using the analytical approximation for random near-optimal cascades. We consider a cascade of length *n* = 5 with a step-function input *R* = 1, *α_i_* = 3 and 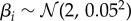. (*a*) Histogram of the asymptotic value given by equation (2.8) using 1000 sampled sets of 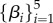 (mean = 7.624, s.d. = 0.043). The solid line marks the mean of the sample and the dashed lines indicate ±2 s.d. (*b*) Histogram of the values obtained using the lower incomplete gamma function in equation (3.2), with 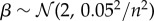 (mean = 7.687, s.d. = 0.039). (*c*) Relationship between the ratio of the variances of the full solution and the gamma function approximation as a function of *n*. (Online version in colour.)

We have tested this idea in figures 3–5. First, we check that cascades with non-identical deactivation rates still achieve maximal amplification when the cascade is of finite length, and we characterize the distribution of cascade lengths observed. Figure 3 shows the histogram of the cascade length at which maximal amplification is achieved for random ensembles of cascades. We consider a step-function input *R*(*t*) = 1 with *α*_1_ = 1.2, and we take as a reference an optimal cascade with identical activation rates *α_i_* = 1 for *i* > 1 and deactivation rates 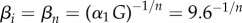, which delivers a gain of *G* = 8 with an optimal finite length of *n* = 4 [4]. We then generate 1000 sets of cascades of length *n* = 1, … ,10, with deactivation rates drawn from a distribution 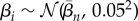, 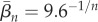, 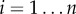 and 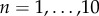 and we record the length at which the maximal amplification occurs. Note that the mean of the deactivation rates depends on the length of the cascade. As shown in figure 3, near-optimal cascades (with normally distributed *β_i_* with mean 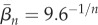) achieve maximal amplification for lengths between *n* = 3 and 5 in 60.4% of cases.

To test whether we can use the gamma function to estimate the parameters of cascades in which the deactivation rates are not identical, we simulated 1000 cascades under a step-function input *R*(*t*) = 1, with *α*_(*n*)_ = 3, *n* = 5, and random deactivation rates 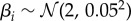. In each cascade, we fitted the parameters 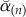,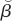,,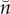 in equation (3.2) to the ‘observed’ 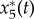. Figure 4 shows the histograms of the fitted parameters for the 1000 random cascades. The fitted parameters are close to their ‘true’ values, with the distributions of 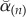 and 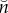 peaked close to their ‘true’ values, and the distribution of estimated deactivation rates 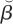 normally distributed around its ‘true’ value.

To check that the outputs for (random) near-optimal cascades can be well approximated using the gamma function expressions, we show in figure 5 that the distribution of asymptotic values of an ensemble of cascades governed by (2.8) with 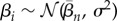 matches the distribution obtained from the gamma function representation (3.2) with 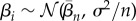. Hence, the gamma function form can be used for near-optimal cascades with random variability in the deactivation parameters, by scaling the variance of the deactivation rates by the length of the cascade (figure 5*c*).

### 4.3. Cascade reordering: lumped representation of identical blocks

As another deviation from strict optimality, we examine how the output of a weakly activated cascade is modified when a single protein in the cascade has a different deactivation rate. For instance, Chaves *et al*. [4] considered an auxiliary protein with different deactivation rate at the end of the cascade. We study the effect of such a ‘perturbation’, and the effect of the position of the perturbation in the cascade.

Consider a cascade of *n* proteins with activation rates *α_j_* and deactivation rates 
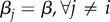
, and 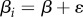 for a given node *i*. First, note that from the Laplace transform of 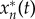, it is clear that the *position in the cascade* of the protein with distinct deactivation *β_i_* does not affect the final output4.1
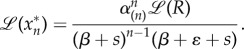


**Figure 6.**
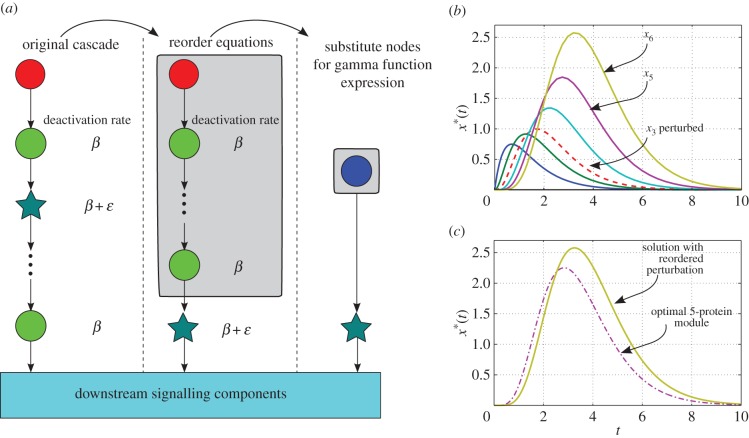
Cascade reordering and lumping of identical protein blocks into sub-cascades. (*a*) A linear *ɛ*-perturbed cascade model; the input (red node) can either be constant or decaying; green circle nodes are proteins whose deactivation rates are all *β*; the blue star node has deactivation rate *β* + *ɛ*. Downstream of the cascade lie other components of the signalling pathway. Reordering of the equations moves the perturbed deactivation to the bottom of the cascade, with no effect on the output of the cascade. The first three equations in the reordered cascade can then be substituted for an incomplete gamma function. (*b*) Time-courses of a six-protein cascade where the third protein has an *ɛ*-perturbed deactivation rate following an exponentially decaying input. (*c*) The dashed-dotted line shows the incomplete gamma function of the re-arranged module of five unperturbed proteins given by equation (3.6), which does *not* correspond to any of the time-courses in (*b*). However, the output of the cascade (i.e. the time course of the perturbed protein moved to the bottom of the cascade given by equation (4.5) and shown as a solid line) coincides with the output of (*b*). (Online version in colour.)

This fact allows us to reshuffle the equations of linear cascade models, grouping the blocks with identical deactivation rates, which can thus be lumped upstream in the cascade and replaced with the incomplete gamma function representation. The equations of the perturbed proteins can be placed downstream and take the gamma function of the lumped block as an input. Such reordering can be used to reduce and simplify the model of a cascade without altering the dynamics or timescales (figure 6*a*).

More explicitly, suppose we have an *ɛ*-perturbed cascade of (*n* + 1) proteins reordered so that the first *n* proteins all have deactivation rate *β* and the (*n* + 1)th protein has rate *β* *+* *ɛ*. For a step-function input *R*(*t*) = 1, *t* ≥ 0 we use equation (3.2) to summarize the first *n* equations, and the equation for the perturbed (*n* + 1)th protein becomes then4.2
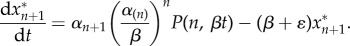
This equation can be solved analytically to give4.3
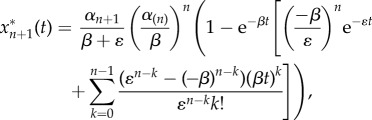
where we have assumed the initial condition 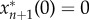.

Likewise, when the input is exponentially decaying 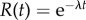, we have that4.4
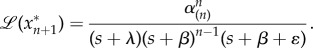
When the initial condition is 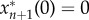, the analytical solution for 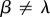 is4.5
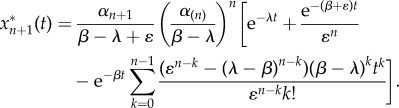
When *λ* = *β* the solution is4.6
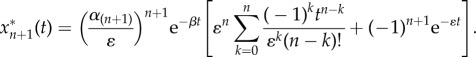


We illustrate these points in figure 6. Figure 6*b* shows the time course of a cascade with six proteins in which the deactivation of the third protein is perturbed. We then reorder the equations so that the perturbed one lies at the bottom. Figure 6*c* shows the output of the first five reordered equations, given by the gamma function expression (3.6) (dot-dashed line), and the analytical solution of the perturbed protein (which is now the output of the cascade, solid line), given by equation (4.5). Note how the time-courses of the fifth protein in the original and rearranged cascades are different, yet the time course of the sixth protein is identical in both cases, as per our solution. Given the results for random cascades presented above, this approach can be applied to lump sub-cascades of proteins with similar deactivation rates which can then be described compactly through their corresponding gamma function modules.

### 4.4. Simplified modules for activation cascades and delay differential equation models

Experimental observations in signalling cascades are typically concerned with the amplification, distortion and delay introduced in the output. As discussed above, when using ODE models, delays are usually incorporated through the addition of extra equations (and their corresponding extra variables and parameters) corresponding to unmeasured, hidden components, steps or processes in the cascade [22]. This approach can lead to large (high-dimensional) models with many unobservable variables and high numbers of parameters to be identified or fitted [23,24]. Alternatively, modellers often use DDEs to account for the lag between an event and its effect [25–27]. In a DDE, the activity of a variable depends on the state of the system a time *τ* in the past:
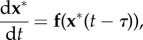
where the parameter *τ* ≥ 0 is the delay. Although linear systems of DDEs can in principle be solved analytically using infinite series involving the Lambert function [28,29], such solutions are often impractical to use.

**Figure 7.**
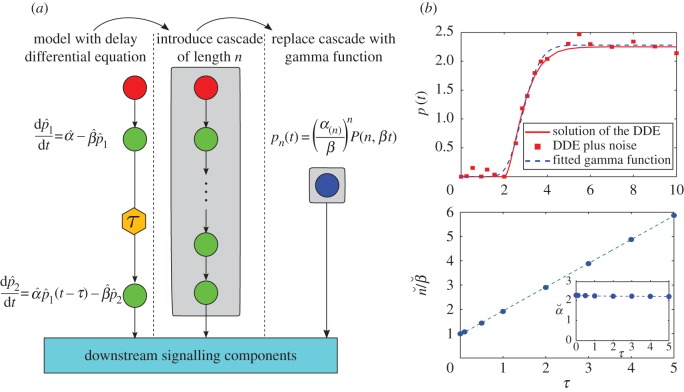
Using linear activation cascades to replace DDEs. (*a*) An input signal (red node) activates a node in a signalling pathway. The bottom node responds with a delay *τ*. The delay in the equation can be substituted with a linear cascade of unknown length *n*, which in turn can be described by a lower incomplete gamma function. (*b*)(i) The solid line is the full solution to the DDE (4.7) and the squares are ‘data points’ taken from this solution with added random noise. The dashed line is the fit of the data using a lower incomplete gamma function. (ii) The ratio of the fitted 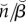 scales linearly with *τ*, the delay in the original data: 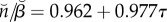 (dashed line). Inset: the fitted parameter 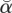 remains constant with varying *τ*. (Online version in colour.)

We have checked that our results can be applied to model simple delays in linear activation cascades, leading to concise ODE models that capture the delay through the gamma function terms without the need to rely on DDEs (figure 7*a*). As an example, consider a system with delay modelled with the linear DDE:4.7
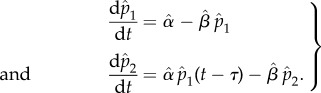

Figure 7*b*(i) shows the simulated time course of 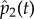 (solid line) when 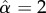, 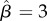 and *τ* = 2 with initial conditions 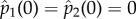. This series was numerically obtained with the dde23 solver in Matlab. We then generate our ‘observed data’ by sampling 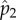 at various time points and adding observational random noise from a distribution 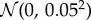.

We then fit this noisy data to our gamma function expression (3.2):4.8
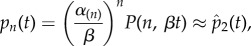
and we estimate the corresponding parameters. Figure 7*b*(i) shows the fit, as obtained with the squeeze-and-breathe algorithm [20], with estimated parameters 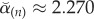, 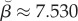 and 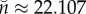.

To explore the connection between the parameters of the DDE and the best-fit activation cascade model, we simulate the DDE (4.7) with parameters 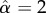 and 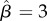 for different values of the delay 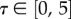 and fit models as above. The dependence of the fitted parameters and *τ* is shown in figure 7*b*(ii). Reassuringly, the amplification factor 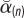 remains relatively constant, whereas the ratio 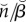 grows linearly with *τ*. This can be expected from the simple argument that the time delay *τ* in the DDE should be related to the accumulated time needed to traverse *n* sequential steps with duration 1/*β*. Hence, a DDE with delay *τ* can be approximated with a linear cascade, by tuning *both* the length and the deactivation rate of the cascade, i.e. 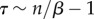.

In Beguerisse-Díaz *et al*. [30], we have used the approach described here to introduce delays in the antioxidant responses of guard cells to abscisic acid and ethylene stimuli during stomatal closure in an ODE model.

## 5. Discussion

In this work, the classic model of activation cascades in the weakly activated regime [1] has been re-examined. We have considered the important case where all deactivation rates of the components of the cascade are identical, which was shown to provide optimal amplification in Chaves *et al*. [4]. Our results show that the output of optimal cascades can be represented exactly by lower incomplete gamma functions, and we show numerically that even when the cascades are near optimal (i.e. when the deactivation rates are iid normal random variables) a gamma function can summarize the cascade by an appropriate rescaling of the parameters. We also show that the position of a protein in the cascade does not affect the final output, so that blocks of proteins with identical deactivation rates can be lumped and represented with incomplete gamma functions. These results allow the reduction of the number of equations and parameters in ODE models without affecting the dynamics or the time scales of the system. We have also shown that in some cases incomplete gamma functions can be used to model delays within systems of ODEs, as an alternative to DDEs.

Beyond its application to enzymatic activation cascades, similar mathematical models of cascades could be helpful for the parametrization and modelling of multi-step transcriptional processes, an area of active research in systems and synthetic biology [16,31–33]. In general, model reduction of systems of differential equations remains a challenging and active area of research [34–36]. Some methods reduce network models (or modules) based on the topology, effectively finding a minimal kernel that preserves some aspects of the dynamics [37]. Yet, by only considering the topology of the system such methods cannot be guaranteed to preserve time scales or behaviour [38], and are best suited for Boolean models. As Beguerisse-Díaz *et al*. [30] show, time scales and transients can be crucially linked to the behaviour of a model and cannot be ignored in many cases. Our work introduces a simplified, compact description that can serve to consider delays in ODE models for systems and synthetic biology, and to fit data from experimental observations.

## Endnotes

1 We used the Matlab command gammainc to evaluate the lower incomplete gamma function.

2 Code available from http://people.maths.ox.ac.uk/beguerisse/.

## Data accessibility

No new data was generated during the course of this research.
